# Supplementary material for: Synthesis, Characterization, and In Vivo Study of Some Novel 3,4,5-Trimethoxybenzylidene-hydrazinecarbothioamides and Thiadiazoles as Anti-Apoptotic Caspase-3 Inhibitors
Source: Molecules. 2022 Mar 31;27(7):2266. doi: 10.3390/molecules27072266 (PMC9040718; doi:10.3390/molecules27072266)
Supplement: Supplementary file 1 [file molecules-27-02266-s001.zip › molecules-1656603-SI.pdf]

molecules

MDPI

Supplementary File

Synthesis, Characterization, and *In Vivo* Study of Some Novel 3,4,5–Trimethoxybenzylidene–hydrazinecarbothioamides and Thiadiazoles as Anti–Apoptotic Caspase–3 Inhibitors

Sara M. Mostafa [1], Ashraf A. Aly [1,*], Stefan Bräse [2,3,*], Martin Nieger [4], Sara Mohamed Naguib Abdelhafez [5], Walaa Yehia Abdelzaher [6] and El–Shimaa M. N. Abdelhafez [7]

1 Chemistry Department, Faculty of Science, Minia University, El–Minia 61519, Egypt; sara.ahmed@mu.edu.eg
2 Institute of Organic Chemistry, Karlsruhe Institute of Technology, 76131 Karlsruhe, Germany
3 Institute of Biological and Chemical Systems (IBCS–FMS), Karlsruhe Institute of Technology, Eggenstein–Leopoldshafen, 76131 Karlsruhe, Germany
4 Department of Chemistry, University of Helsinki, P.O. Box 55 (A. I. Virtasen aukio I), 00014 Helsinki, Finland; martin.nieger@helsinki.fi.
5 Department of Histology, Faculty of Medicine, Minia University, El–Minia 61519, Egypt; sara_histology@yahoo.com.
6 Department of Pharmacology, Faculty of Medicine, Minia University, El–Minia 61519, Egypt; walaayehia22@yahoo.com.
7 Department of Medicinal Chemistry, Faculty of Pharmacy, Minia University, El–Minia 61519, Egypt; shimaanaguib_80@yahoo.com.
* Correspondence: ashraf.shehata@mu.edu.eg (A.A.A.), stefan.braese@kit.edu (S.B.)

Single crystal X-ray structure determination of 2d

Single crystals were obtained by recrystallization from acetonitrile. The single crystal X-ray diffraction study was carried out on an Bruker D8 VENTURE diffractometer with PhotonII CPAD detector at 298 K using Cu *K* radiation (= 1.54178 Å). Direct methods (SHELXS-98) were used for structure solution and refinement was carried out using SHELXL-2013[42] (full-matrix least-squares on F^2).

2d: $C_{18}H_{19}N_5O_3S$, M = 385.44 g mol^{-1}, yellow crystal, size 0.32 × 0.08 × 0.02 mm, monoclinic space group $P2_1/n$ (no.14), a = 17.8658 (3) Å, b = 5.3161(2) Å, c = 19.6971 (3) Å, β = 96.408 (1) Å, V = 1860.99 (5) Å^3, Z = 4, D_{calcd} = 1.376 mg m^{-3}, F(000) = 808, μ = 1.80 mm^{-1}, T = 298 K, 15205 measured reflection ($2\theta_{max}$ = 72.4^0), 3683 independent [R_{int} = 0.031], 253 parameters, 2 restraint, R_1 [for 3428 $I > 2\sigma(1)$] = 0.032, wR^2 (for all data) = 0.092, S = 1.07, largest diff. peak and hole = 0.22 eÅ^{-3}/- 0.23 eÅ^{-3}.

Crystallographic data (excluding structure factors) for the structure reported in this work has been deposited with Cambridge crystallographic Data Center on supplementary publication no. CCDC-2056821 Copies of the data can be obtained free of charge on application to the director, CCDC, 12 Union Road, Cambridge CB2 IEZ, UK (Fax: +44(1223)3360333: e-mail: deposit@ccdc.cam.ac.uk.

Table S1. Crystal data of **2d.**

$C_{18}H_{19}N_5O_3S$	$F(000) = 808$
$M_r = 385.44$	$D_x = 1.376$ Mg m^{-3}
Monoclinic, $P2_1/n$ *(no.14)*	Cu K radiation, = 1.54178 Å
$a = 17.8658$ (3) Å	Cell parameters from 9954 reflections
$b = 5.3216$ (1) Å	= 3.5–72.3°
$c = 19.6971$ (3) Å	= 1.80 mm^{-1}
= 96.408 (1)°	$T = 298$ K
$V = 1860.99$ (5) Å^3	Plates, yellow
$Z = 4$	0.32 × 0.08 × 0.02 mm

Table S2. Data collection of **2d.**

Bruker D8 VENTURE diffractometer with Photo-nII CPAD detector	3428 reflections with $I > 2$ (I)
Radiation source: INCOATEC microfocus sealed tube	$R_{int} = 0.031$
rotation in and , 1°, shutterless scans	$_{max}$ = 72.4°, $_{min}$ = 3.5°
Absorption correction: multi-scan *SADABS* (Sheldrick, 2014)	h = -22 22
$T_{min} = 0.732$, $T_{max} = 0.958$	k = -6 6
15205 measured reflections	l = -24 24
3683 independent reflections	

Table S3. Refinement of **2d.**

Refinement on F^2	Primary atom site location: dual
Least-squares matrix: full	Secondary atom site location: difference Fourier map
$R[F^2 > 2 (F^2)] = 0.032$	Hydrogen site location: difference Fourier map
$wR(F^2) = 0.092$	H atoms treated by a mixture of independent and constrained refinement
$S = 1.07$	$w = 1/[^2(F_o^2) + (0.0475P)^2 + 0.3754P]$ where $P = (F_o^2 + 2F_c^2)/3$
3683 reflections	(/)$_{max}$ = 0.001
253 parameters	$_{max}$ = 0.22 e Å^{-3}
2 restraints	$_{min}$ = -0.23 e Å^{-3}

Computing details

Data collection: *APEX3* (Bruker AXS Inc., 2016); cell refinement: *APEX3* (Bruker AXS Inc., 2016); data reduction: *SAINT* (Bruker AXS Inc., 2016); program(s) used to solve structure: SHELXT (Sheldrick, 2015); program(s) used to refine structure: *SHELXL2014*/7 (Sheldrick, 2014); software used to prepare material for publication: *publCIF* (Westrip, 2010).

Special details

Experimental. dx = 40 mm, 1 deg., 8+1 runs, 1374 frames, 30/120 sec./frame
Geometry. All esds (except the esd in the dihedral angle between two l.s. planes) are estimated using the full covariance matrix. The cell esds are taken into account individually in the estimation of esds in distances, angles and torsion angles; correlations between esds in cell parameters are only used when they are defined by crystal symmetry. An approximate (isotropic) treatment of cell esds is used for estimating esds involving l.s. planes.

Table S4: Fractional atomic coordinates and isotropic or equivalent isotropic displacement parameters (Å^2) of **2d**

	X	*Y*	*Z*	U_{iso}*/U_{eq}
S1	0.64398 (2)	0.47208 (6)	0.57098 (2)	0.04273 (11)
C2	0.63911 (7)	0.5691 (2)	0.48518 (6)	0.0379 (3)
N3	0.59028 (6)	0.4517 (2)	0.44365 (5)	0.0404 (2)
N4	0.55292 (6)	0.2680 (2)	0.47737 (5)	0.0405 (2)
C5	0.57500 (7)	0.2561 (2)	0.54248 (6)	0.0360 (3)
N6	0.54868 (7)	0.0862 (2)	0.58462 (5)	0.0437 (3)
H6	0.5170 (9)	-0.024 (3)	0.5699 (9)	0.052*
N7	0.57461 (6)	0.1013 (2)	0.65213 (5)	0.0381 (2)
C8	0.54873 (7)	-0.0614 (2)	0.69127 (6)	0.0389 (3)
H8	0.5141	-0.1808	0.6731	0.047*
C9	0.57373 (7)	-0.0597 (2)	0.76464 (6)	0.0371 (3)
C10	0.62827 (7)	0.1103 (2)	0.79194 (6)	0.0390 (3)
H10	0.6490	0.2248	0.7637	0.047*
C11	0.65135 (7)	0.1079 (2)	0.86141 (6)	0.0379 (3)
O11	0.70435 (5)	0.26792 (19)	0.89285 (5)	0.0476 (2)
C111	0.73895 (9)	0.4384 (3)	0.84987 (9)	0.0573 (4)
H11A	0.7642	0.3453	0.8174	0.086*
H11B	0.7748	0.5407	0.8773	0.086*
H11C	0.7011	0.5438	0.8260	0.086*
C12	0.62021 (7)	-0.0643 (2)	0.90426 (6)	0.0402 (3)
O12	0.64723 (6)	-0.0791 (2)	0.97235 (5)	0.0532 (3)
C121	0.61729 (9)	0.1034 (3)	1.01457 (7)	0.0557 (4)
H12A	0.6237	0.2680	0.9961	0.084*
H12B	0.6433	0.0939	1.0599	0.084*
H12C	0.5646	0.0715	1.0162	0.084*
C13	0.56438 (7)	-0.2290 (2)	0.87664 (6)	0.0401 (3)
O13	0.53532 (6)	-0.3848 (2)	0.92223 (5)	0.0532 (3)
C131	0.47514 (10)	-0.5459 (3)	0.89672 (9)	0.0587 (4)
H13A	0.4355	-0.4479	0.8728	0.088*
H13B	0.4563	-0.6321	0.9341	0.088*
H13C	0.4930	-0.6665	0.8661	0.088*
C14	0.54171 (7)	-0.2289 (2)	0.80673 (6)	0.0406 (3)
H14	0.5052	-0.3418	0.7882	0.049*
N15	0.68606 (7)	0.7589 (2)	0.46933 (6)	0.0486 (3)
H15	0.7204 (9)	0.799 (3)	0.5006 (8)	0.058*
C16	0.68676 (7)	0.8860 (2)	0.40703 (7)	0.0407 (3)
C17	0.63880 (8)	0.8289 (3)	0.34883 (7)	0.0483 (3)
H17	0.6043	0.6982	0.3493	0.058*
C18	0.64279 (9)	0.9692 (3)	0.28974 (8)	0.0554 (4)
H18	0.6103	0.9323	0.2508	0.067*
C19	0.69388 (10)	1.1612 (3)	0.28784 (8)	0.0589 (4)
H19	0.6961	1.2534	0.2480	0.071*
C20	0.74151 (10)	1.2152 (3)	0.34551 (9)	0.0611 (4)

H20	0.7764	1.3446	0.3445	0.073*
C21	0.73856 (9)	1.0805 (3)	0.40517 (8)	0.0520 (3)
H21	0.7711	1.1197	0.4439	0.062*

Table S5: Atomic displacement parameters (Å^2) of **2d**

	U^{11}	U^{22}	U^{33}	U^{12}	U^{13}	U^{23}
S1	0.0483 (2)	0.04497 (19)	0.03236 (17)	-0.01106 (13)	-0.00701 (13)	0.00637 (12)
C2	0.0385 (6)	0.0408 (6)	0.0333 (6)	-0.0013 (5)	-0.0004 (5)	0.0060 (5)
N3	0.0427 (6)	0.0458 (6)	0.0322 (5)	-0.0080 (5)	0.0016 (4)	0.0057 (4)
N4	0.0453 (6)	0.0451 (6)	0.0300 (5)	-0.0103 (5)	-0.0004 (4)	0.0048 (4)
C5	0.0375 (6)	0.0387 (6)	0.0309 (6)	-0.0017 (5)	-0.0001 (5)	0.0022 (5)
N6	0.0524 (6)	0.0491 (6)	0.0276 (5)	-0.0163 (5)	-0.0040 (4)	0.0053 (4)
N7	0.0412 (5)	0.0445 (6)	0.0273 (5)	-0.0045 (4)	-0.0024 (4)	0.0027 (4)
C8	0.0402 (6)	0.0450 (7)	0.0306 (6)	-0.0070 (5)	-0.0003 (5)	0.0025 (5)
C9	0.0381 (6)	0.0430 (6)	0.0295 (6)	-0.0003 (5)	0.0014 (5)	0.0035 (5)
C10	0.0403 (6)	0.0436 (7)	0.0328 (6)	-0.0021 (5)	0.0026 (5)	0.0039 (5)
C11	0.0352 (6)	0.0420 (6)	0.0354 (6)	0.0039 (5)	-0.0015 (5)	-0.0020 (5)
O11	0.0434 (5)	0.0549 (6)	0.0421 (5)	-0.0046 (4)	-0.0062 (4)	-0.0045 (4)
C111	0.0512 (8)	0.0555 (9)	0.0632 (9)	-0.0125 (7)	-0.0024 (7)	-0.0016 (7)
C12	0.0454 (7)	0.0458 (7)	0.0282 (6)	0.0101 (5)	-0.0015 (5)	0.0028 (5)
O12	0.0663 (6)	0.0610 (6)	0.0295 (5)	0.0161 (5)	-0.0066 (4)	0.0032 (4)
C121	0.0676 (9)	0.0648 (9)	0.0341 (7)	0.0018 (7)	0.0030 (6)	-0.0026 (6)
C13	0.0458 (7)	0.0418 (7)	0.0331 (6)	0.0056 (5)	0.0062 (5)	0.0078 (5)
O13	0.0664 (6)	0.0566 (6)	0.0372 (5)	-0.0049 (5)	0.0087 (4)	0.0149 (4)
C131	0.0659 (10)	0.0562 (9)	0.0560 (9)	-0.0080 (7)	0.0158 (7)	0.0132 (7)
C14	0.0426 (6)	0.0441 (7)	0.0347 (6)	-0.0042 (5)	0.0022 (5)	0.0045 (5)
N15	0.0483 (6)	0.0548 (7)	0.0402 (6)	-0.0168 (5)	-0.0059 (5)	0.0109 (5)
C16	0.0407 (6)	0.0406 (6)	0.0413 (7)	0.0006 (5)	0.0068 (5)	0.0060 (5)
C17	0.0478 (7)	0.0536 (8)	0.0433 (7)	-0.0062 (6)	0.0033 (6)	0.0101 (6)
C18	0.0582 (9)	0.0663 (10)	0.0416 (7)	0.0011 (7)	0.0045 (6)	0.0113 (7)
C19	0.0731 (10)	0.0551 (9)	0.0513 (8)	0.0017 (8)	0.0191 (8)	0.0169 (7)
C20	0.0751 (10)	0.0490 (8)	0.0619 (9)	-0.0151 (8)	0.0190 (8)	0.0077 (7)
C21	0.0588 (8)	0.0478 (8)	0.0494 (8)	-0.0113 (7)	0.0063 (7)	0.0027 (6)

Table S6: Geometric parameters (Å, º) for **2d**

S1—C5	1.7321 (12)	O12—C121	1.4214 (19)
S1—C2	1.7602 (12)	C121—H12A	0.9600
C2—N3	1.2889 (17)	C121—H12B	0.9600
C2—N15	1.3708 (17)	C121—H12C	0.9600
N3—N4	1.3932 (14)	C13—O13	1.3665 (15)
N4—C5	1.3004 (15)	C13—C14	1.3913 (17)
C5—N6	1.3471 (16)	O13—C131	1.422 (2)
N6—N7	1.3606 (14)	C131—H13A	0.9600
N6—H6	0.844 (14)	C131—H13B	0.9600
N7—C8	1.2800 (16)	C131—H13C	0.9600

C8—C9	1.4641 (16)	C14—H14	0.9300
C8—H8	0.9300	N15—C16	1.4023 (17)
C9—C14	1.3899 (17)	N15—H15	0.847 (14)
C9—C10	1.3930 (18)	C16—C17	1.3862 (19)
C10—C11	1.3845 (17)	C16—C21	1.3916 (19)
C10—H10	0.9300	C17—C18	1.391 (2)
C11—O11	1.3692 (15)	C17—H17	0.9300
C11—C12	1.4022 (19)	C18—C19	1.374 (2)
O11—C111	1.4278 (19)	C18—H18	0.9300
C111—H11A	0.9600	C19—C20	1.372 (3)
C111—H11B	0.9600	C19—H19	0.9300
C111—H11C	0.9600	C20—C21	1.383 (2)
C12—O12	1.3760 (15)	C20—H20	0.9300
C12—C13	1.392 (2)	C21—H21	0.9300
C5—S1—C2	85.78 (6)	H12A—C121—H12B	109.5
N3—C2—N15	126.92 (11)	O12—C121—H12C	109.5
N3—C2—S1	115.11 (9)	H12A—C121—H12C	109.5
N15—C2—S1	117.97 (9)	H12B—C121—H12C	109.5
C2—N3—N4	111.35 (10)	O13—C13—C14	124.10 (12)
C5—N4—N3	113.30 (10)	O13—C13—C12	115.68 (11)
N4—C5—N6	123.51 (11)	C14—C13—C12	120.22 (12)
N4—C5—S1	114.44 (9)	C13—O13—C131	117.46 (11)
N6—C5—S1	122.02 (9)	O13—C131—H13A	109.5
C5—N6—N7	117.28 (10)	O13—C131—H13B	109.5
C5—N6—H6	121.5 (12)	H13A—C131—H13B	109.5
N7—N6—H6	121.2 (12)	O13—C131—H13C	109.5
C8—N7—N6	116.24 (10)	H13A—C131—H13C	109.5
N7—C8—C9	119.95 (11)	H13B—C131—H13C	109.5
N7—C8—H8	120.0	C9—C14—C13	119.80 (12)
C9—C8—H8	120.0	C9—C14—H14	120.1
C14—C9—C10	120.48 (11)	C13—C14—H14	120.1
C14—C9—C8	118.96 (11)	C2—N15—C16	128.31 (11)
C10—C9—C8	120.56 (11)	C2—N15—H15	115.6 (12)
C11—C10—C9	119.59 (12)	C16—N15—H15	116.0 (12)
C11—C10—H10	120.2	C17—C16—C21	119.52 (13)
C9—C10—H10	120.2	C17—C16—N15	123.56 (12)
O11—C11—C10	123.76 (12)	C21—C16—N15	116.91 (13)
O11—C11—C12	115.79 (11)	C16—C17—C18	119.31 (14)
C10—C11—C12	120.45 (12)	C16—C17—H17	120.3
C11—O11—C111	116.77 (11)	C18—C17—H17	120.3
O11—C111—H11A	109.5	C19—C18—C17	121.23 (15)
O11—C111—H11B	109.5	C19—C18—H18	119.4
H11A—C111—H11B	109.5	C17—C18—H18	119.4
O11—C111—H11C	109.5	C20—C19—C18	119.08 (14)
H11A—C111—H11C	109.5	C20—C19—H19	120.5

H11B—C111—H11C	109.5	C18—C19—H19	120.5
O12—C12—C13	120.19 (12)	C19—C20—C21	121.08 (15)
O12—C12—C11	120.30 (12)	C19—C20—H20	119.5
C13—C12—C11	119.43 (11)	C21—C20—H20	119.5
C12—O12—C121	114.65 (10)	C20—C21—C16	119.78 (15)
O12—C121—H12A	109.5	C20—C21—H21	120.1
O12—C121—H12B	109.5	C16—C21—H21	120.1
C5—S1—C2—N3	1.20 (11)	C13—C12—O12—C121	-100.94 (15)
C5—S1—C2—N15	-179.25 (11)	C11—C12—O12—C121	82.37 (16)
N15—C2—N3—N4	179.44 (13)	O12—C12—C13—O13	5.37 (18)
S1—C2—N3—N4	-1.05 (15)	C11—C12—C13—O13	-177.91 (11)
C2—N3—N4—C5	0.22 (16)	O12—C12—C13—C14	-174.35 (12)
N3—N4—C5—N6	-177.39 (12)	C11—C12—C13—C14	2.37 (19)
N3—N4—C5—S1	0.72 (14)	C14—C13—O13—C131	-3.8 (2)
C2—S1—C5—N4	-1.04 (10)	C12—C13—O13—C131	176.48 (13)
C2—S1—C5—N6	177.10 (12)	C10—C9—C14—C13	-0.1 (2)
N4—C5—N6—N7	-177.54 (12)	C8—C9—C14—C13	-179.58 (12)
S1—C5—N6—N7	4.49 (17)	O13—C13—C14—C9	178.80 (12)
C5—N6—N7—C8	179.84 (12)	C12—C13—C14—C9	-1.5 (2)
N6—N7—C8—C9	179.80 (12)	N3—C2—N15—C16	7.2 (2)
N7—C8—C9—C14	176.38 (12)	S1—C2—N15—C16	-172.25 (12)
N7—C8—C9—C10	-3.07 (19)	C2—N15—C16—C17	-2.2 (2)
C14—C9—C10—C11	0.89 (19)	C2—N15—C16—C21	177.28 (14)
C8—C9—C10—C11	-179.68 (12)	C21—C16—C17—C18	-0.7 (2)
C9—C10—C11—O11	-179.80 (11)	N15—C16—C17—C18	178.72 (14)
C9—C10—C11—C12	-0.01 (19)	C16—C17—C18—C19	0.7 (2)
C10—C11—O11—C111	-2.54 (18)	C17—C18—C19—C20	-0.2 (3)
C12—C11—O11—C111	177.65 (12)	C18—C19—C20—C21	-0.3 (3)
O11—C11—C12—O12	-5.08 (17)	C19—C20—C21—C16	0.3 (3)
C10—C11—C12—O12	175.11 (12)	C17—C16—C21—C20	0.2 (2)
O11—C11—C12—C13	178.19 (11)	N15—C16—C21—C20	-179.23 (14)
C10—C11—C12—C13	-1.62 (19)		

Table S7: *Hydrogen-bond geometry (Å, º) for* **2d**

D—H···*A*	*D*—H	H···*A*	*D*···*A*	*D*—H···*A*
N6—H6···N4[i]	0.84 (1)	1.96 (1)	2.8010 (15)	172 (2)
C111—H11*B*···S1[ii]	0.96	2.84	3.7608 (15)	160

N15—H15···O11[ii]	0.85 (1)	2.37 (2)	3.1652 (14)	157 (2)
C21—H21···O12[iii]	0.93	2.62	3.4880 (18)	155

Symmetry codes: (i) -*x*+1, -*y*, -*z*+1; (ii) -*x*+3/2, *y*+1/2, -*z*+3/2; (iii) -*x*+3/2, *y*+3/2, -*z*+3/2.

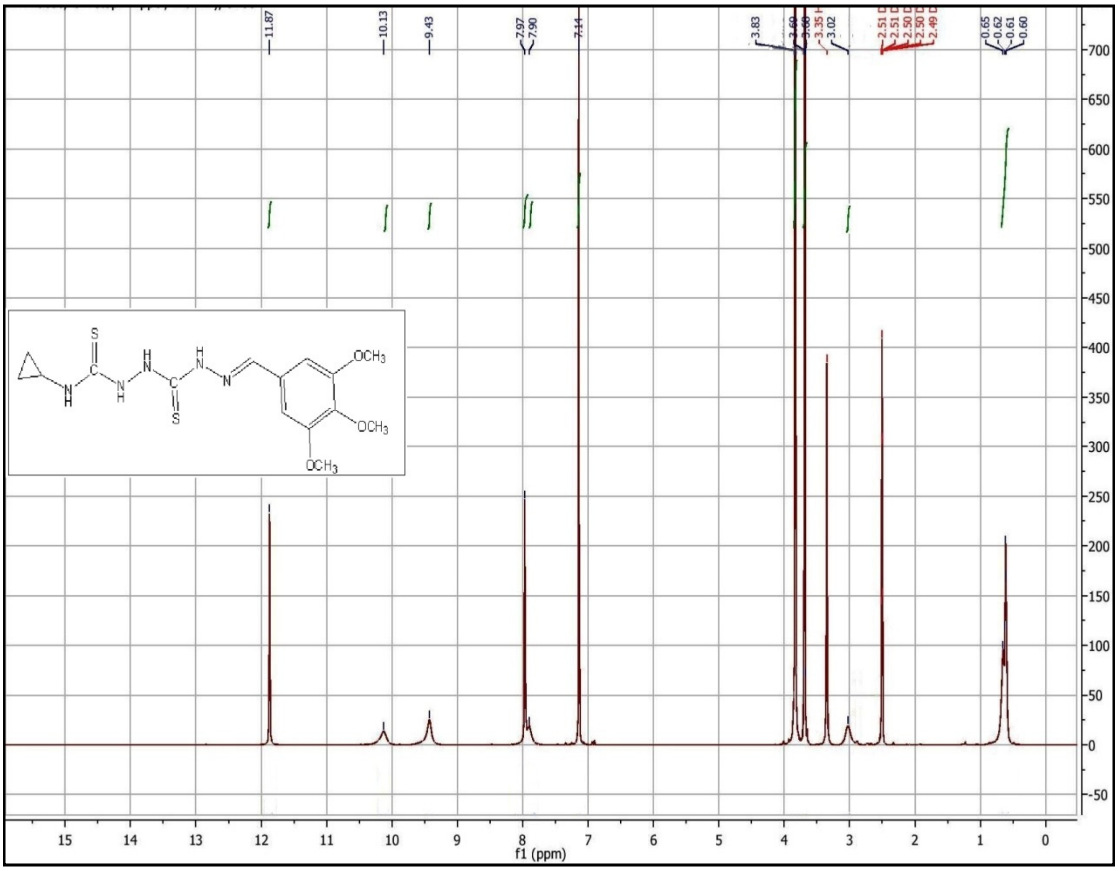

SI Figure S1: ^{1}H-NMR spectrum of **1c**

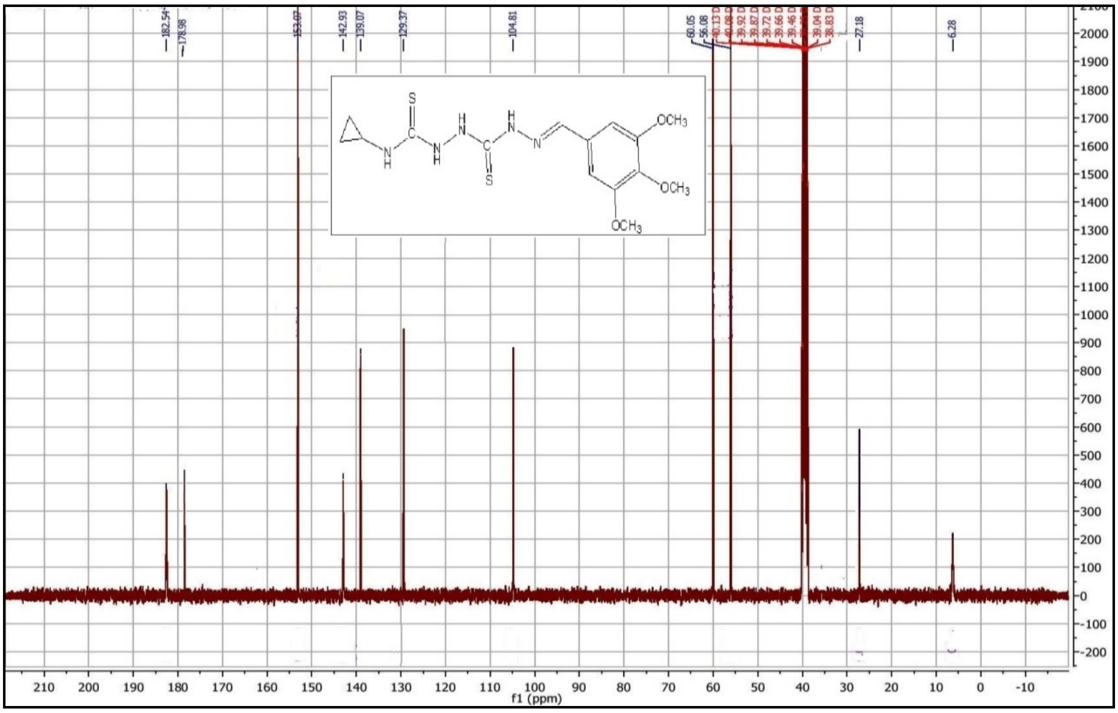

SI Figure S2: ^{13}C-NMR spectrum of **1c**

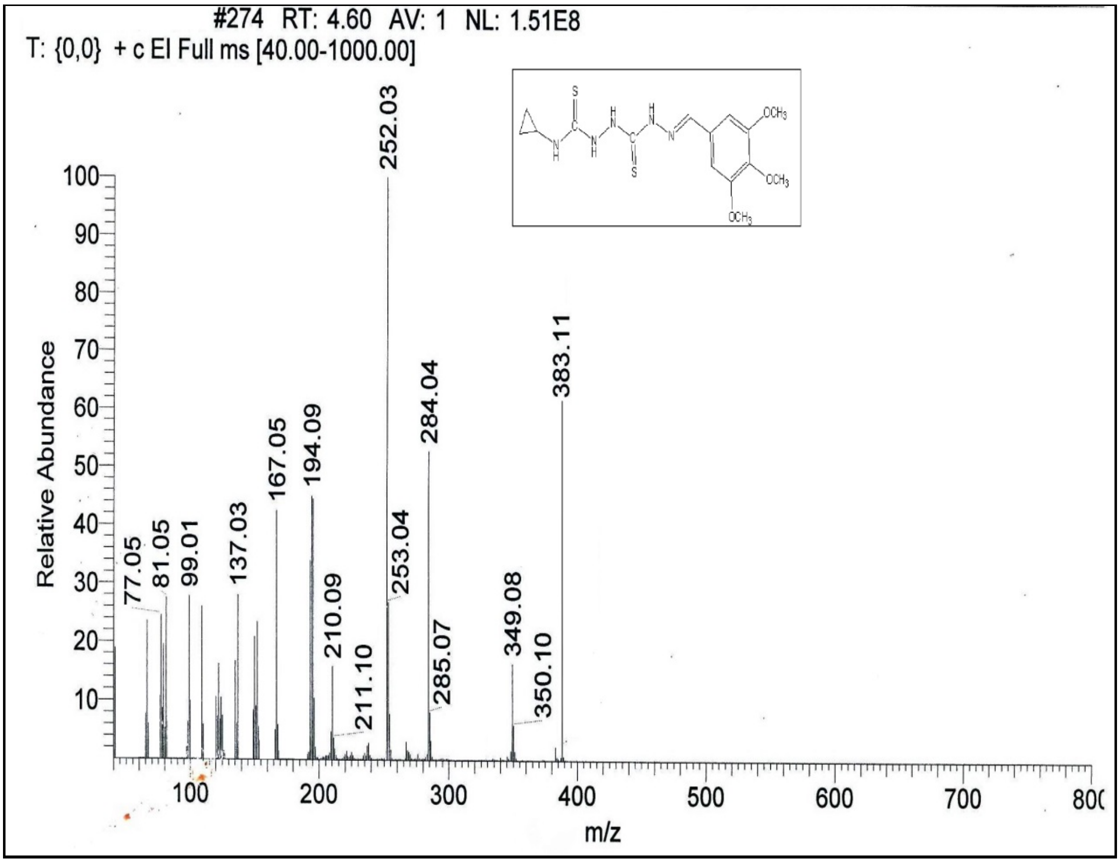

SI Figure S3: Mass spectroscopy of **1c**

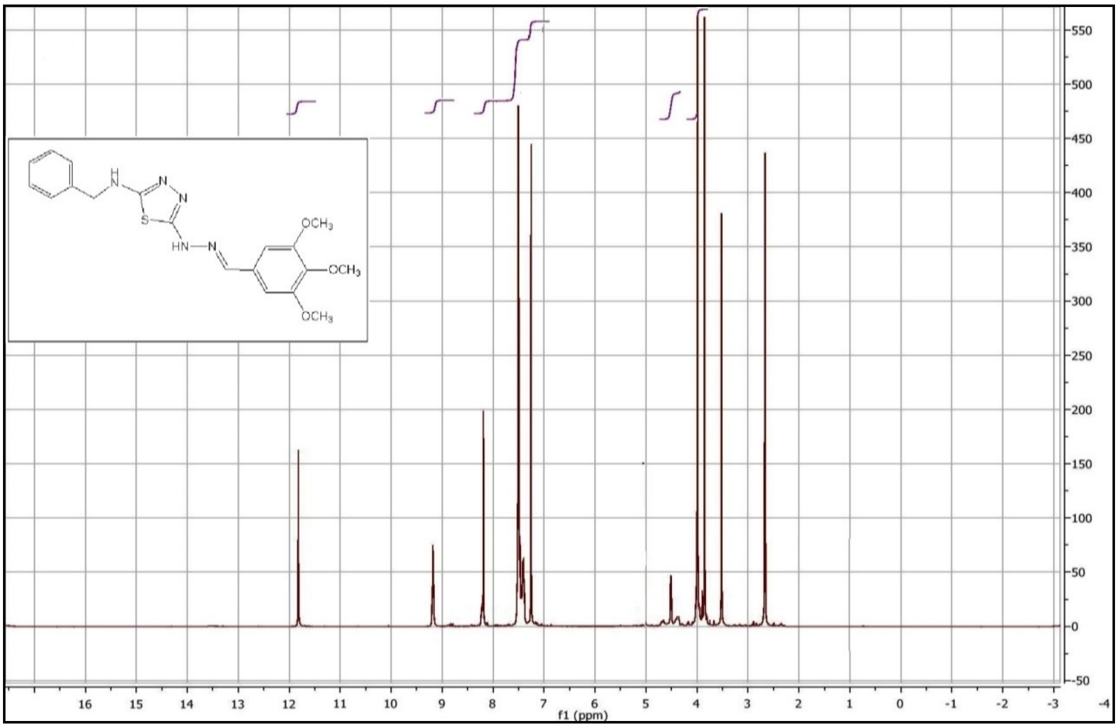

SI Figure S4: ^{1}H-NMR spectrum of **2a**

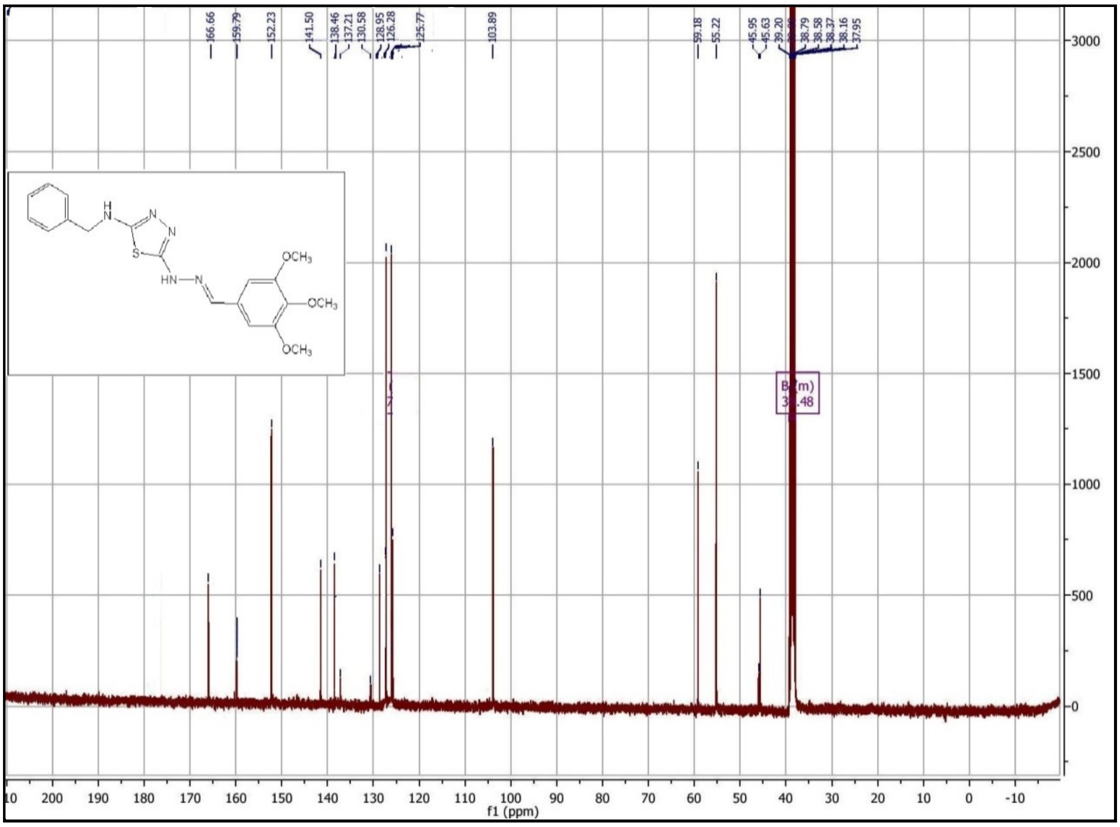

SI Figure S5: ^{13}C-NMR spectrum of **2a**

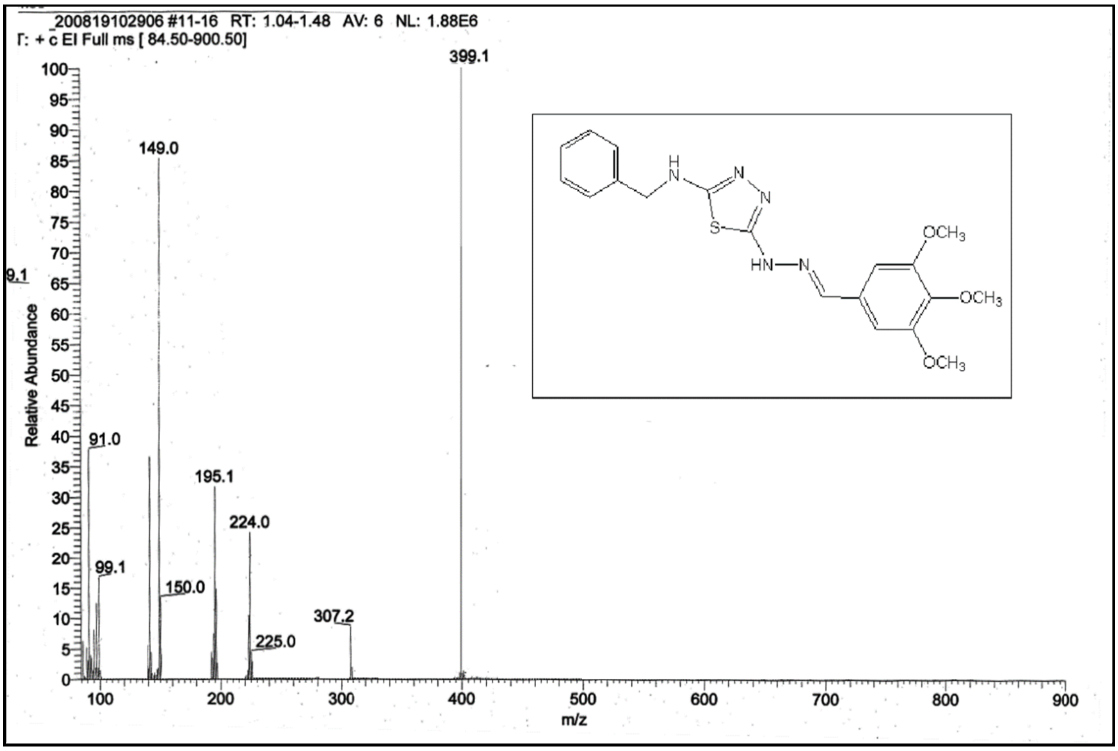

SI Figure S6: Mass spectroscopy of **2a**

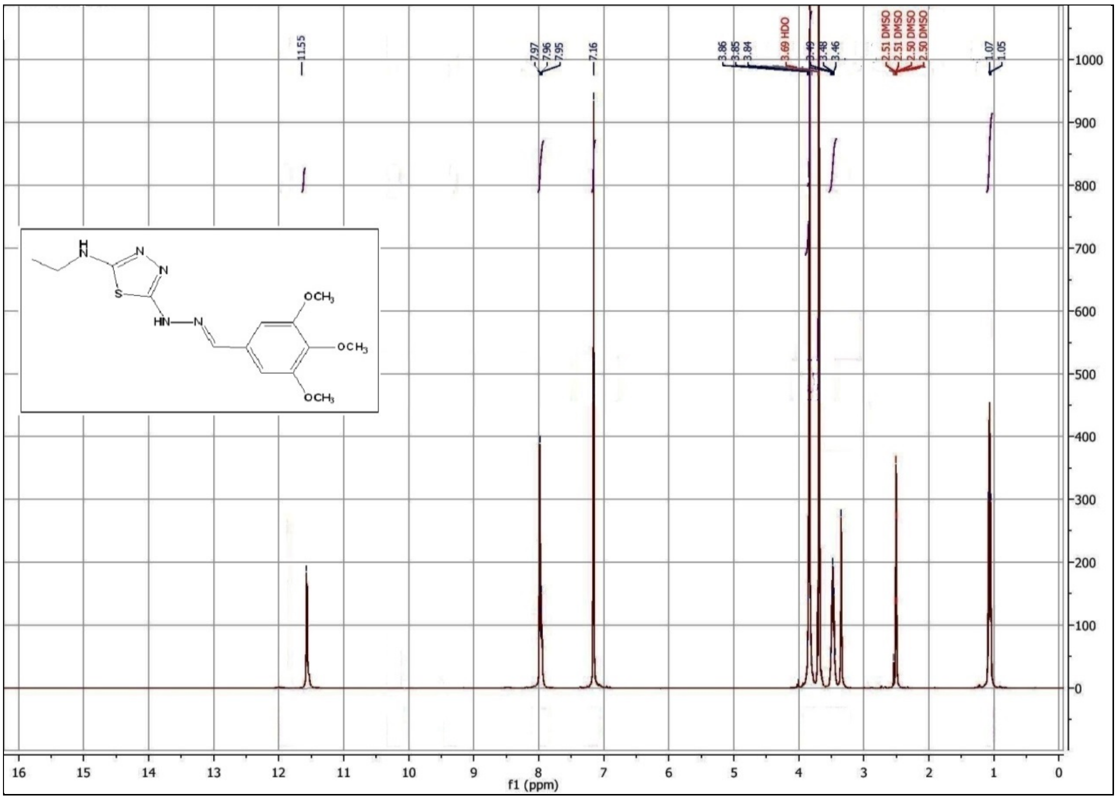

SI Figure S7: ^{1}H-NMR spectrum of **2b**

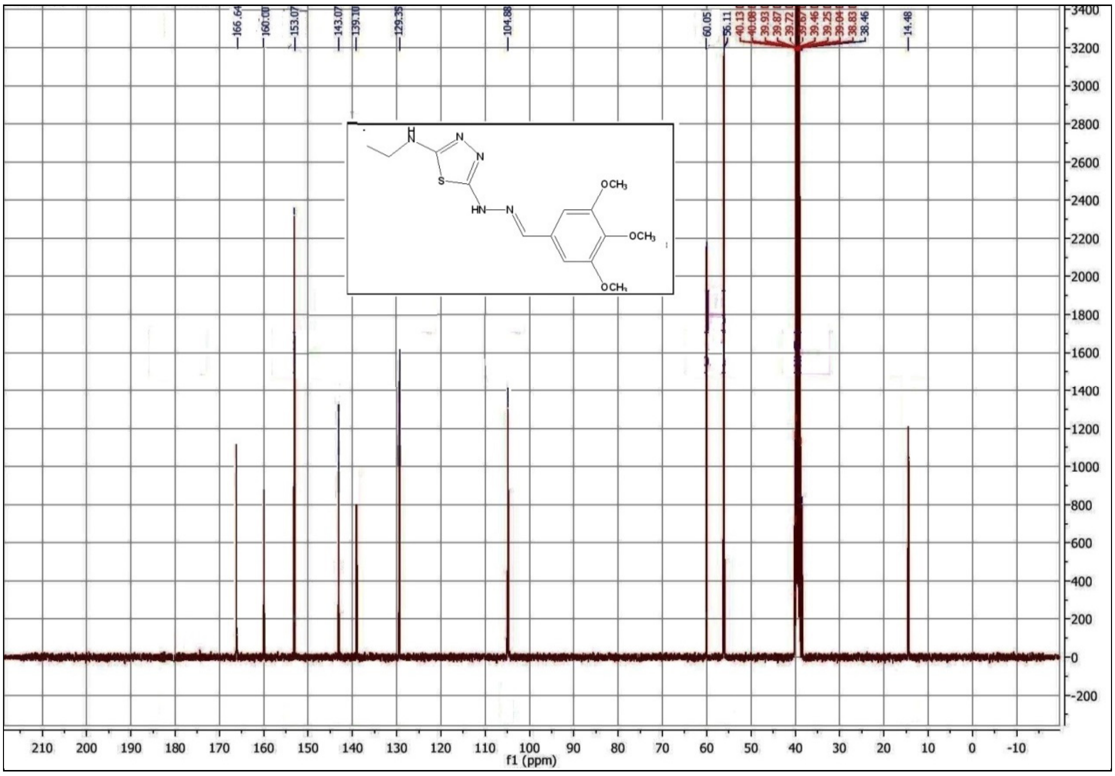

SI Figure S8: ^{13}C-NMR spectrum of **2b**

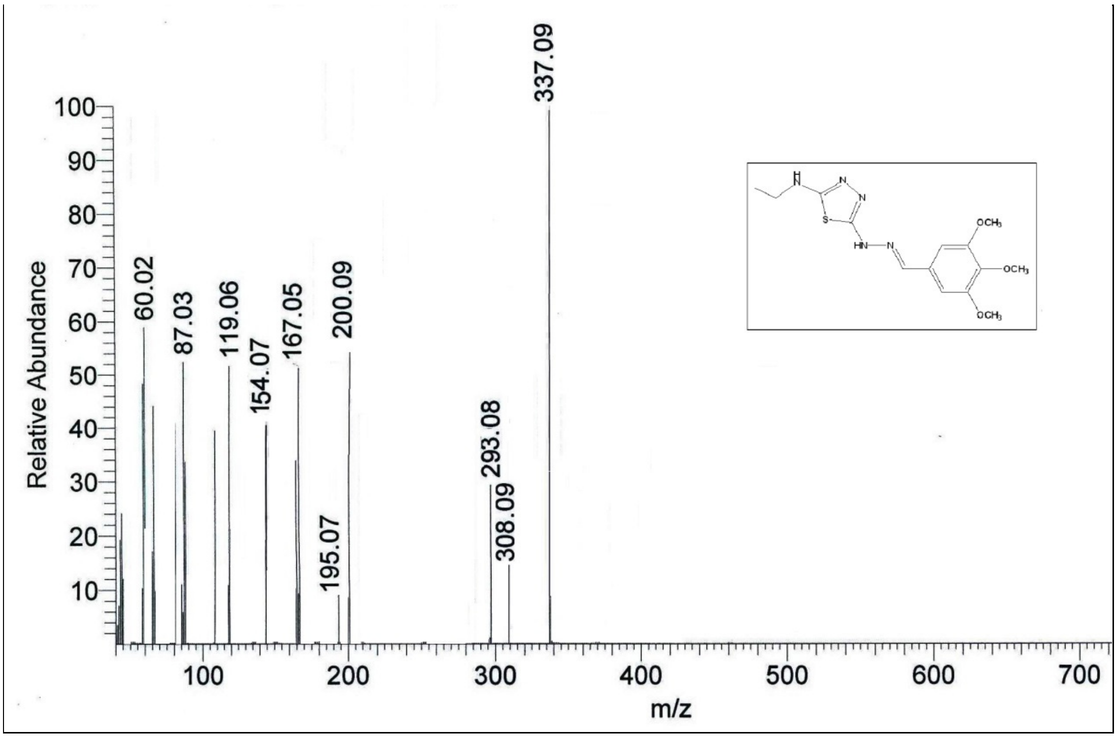

SI Figure S9: Mass spectroscopy of **2b**

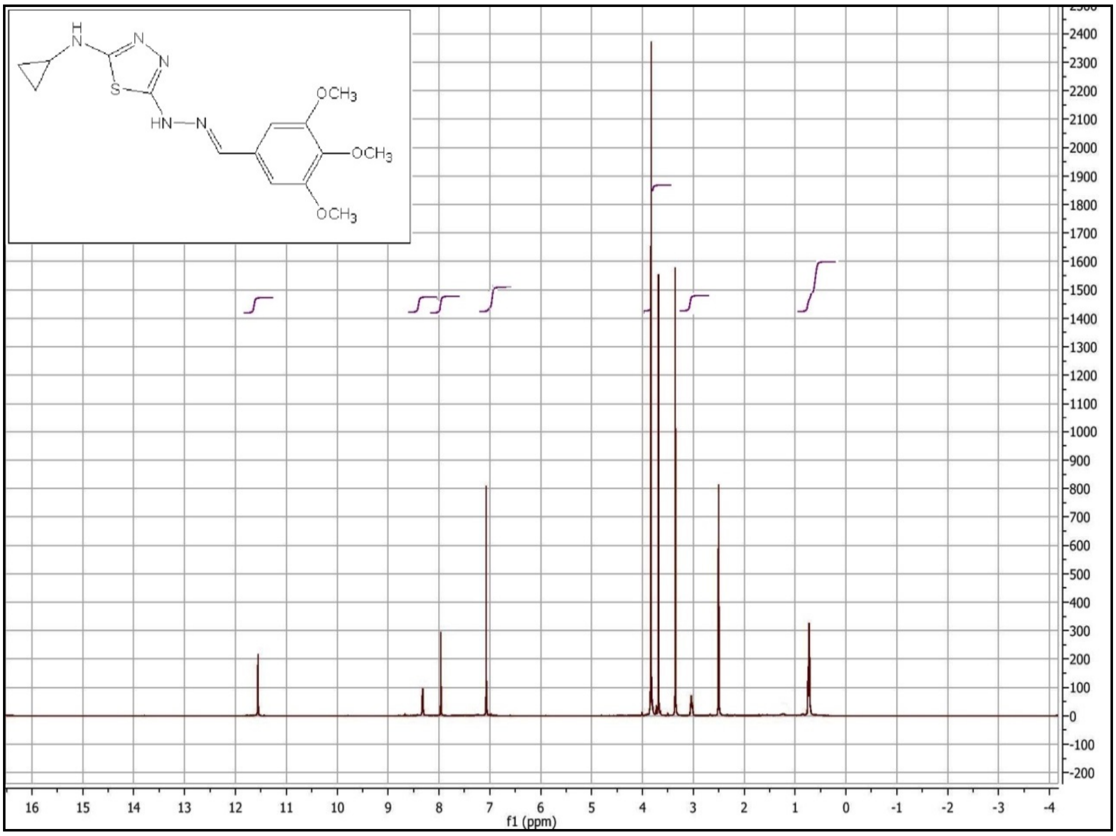

SI Figure S10: ^{1}H-NMR spectrum of **2c**

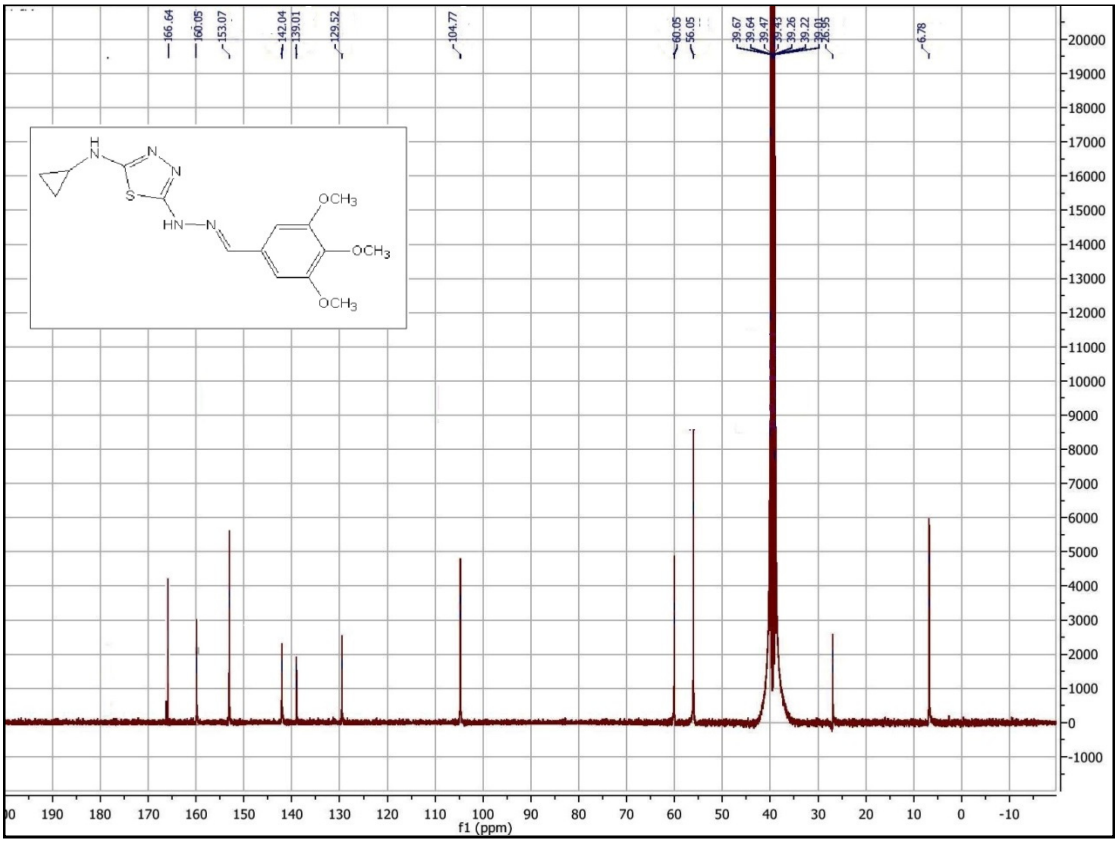

SI Figure S11: ^{13}C-NMR spectrum of **2c**

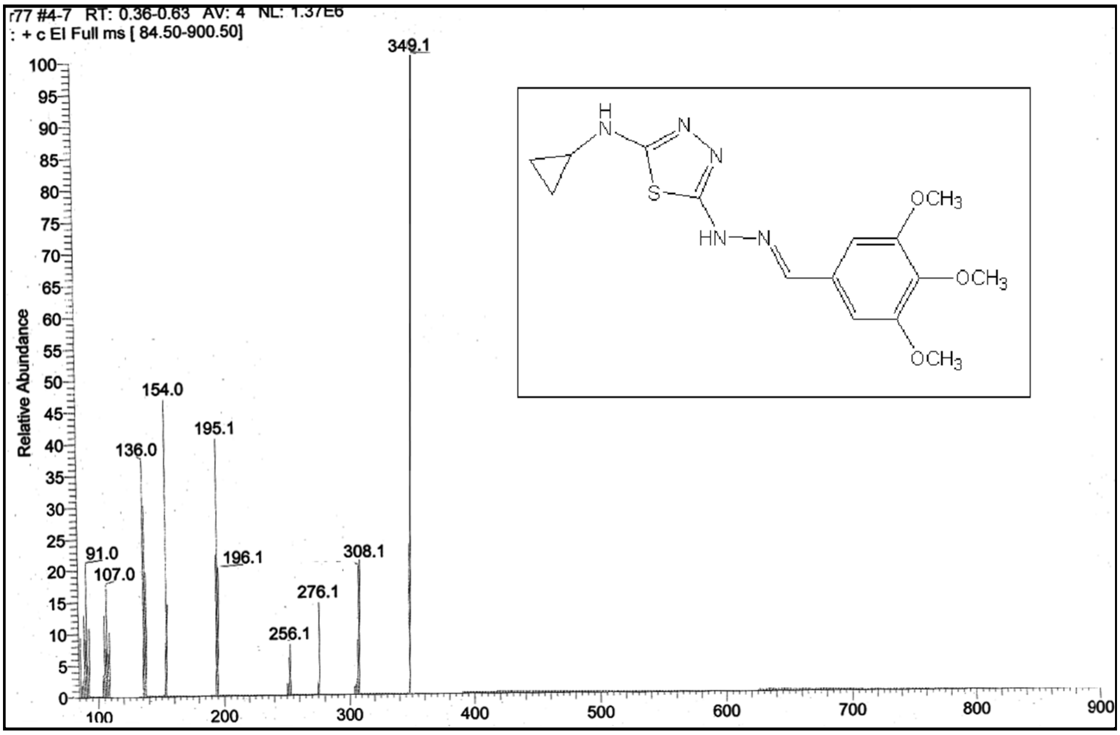

SI Figure S12: Mass spectroscopy of **2c**

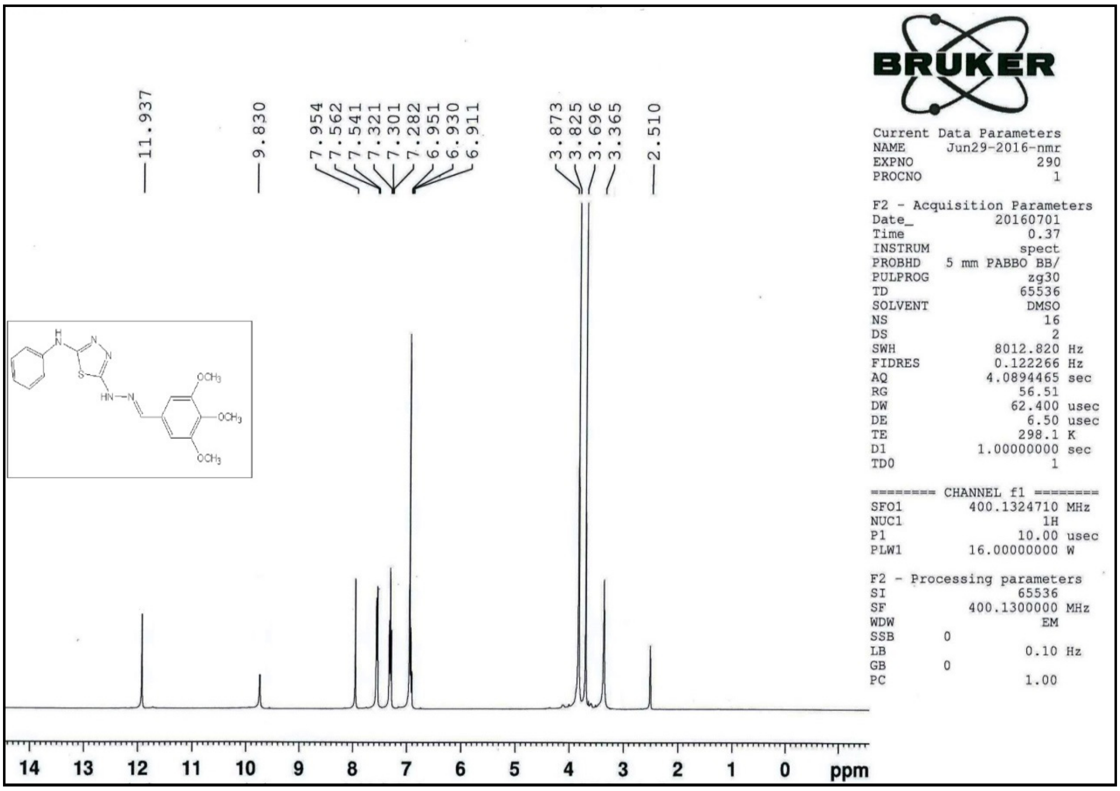

SI Figure S13: ^{1}H-NMR spectrum of **2d**

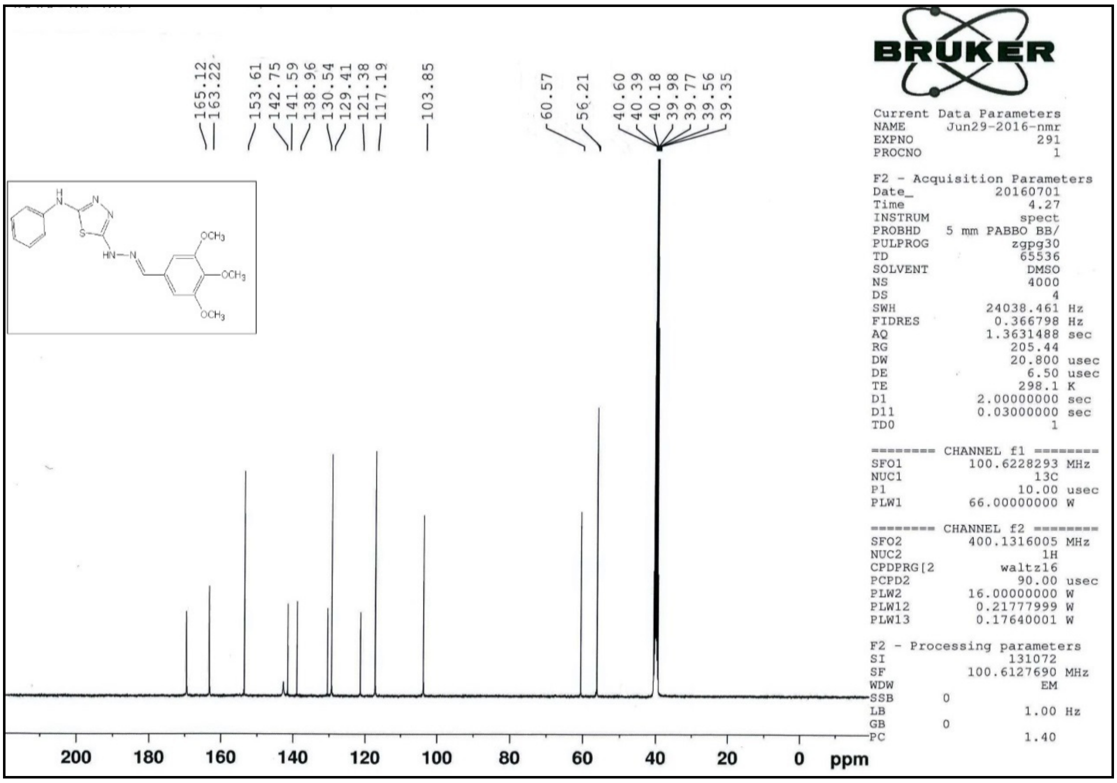

SI Figure S14: ^{13}C-NMR spectrum of **2d**

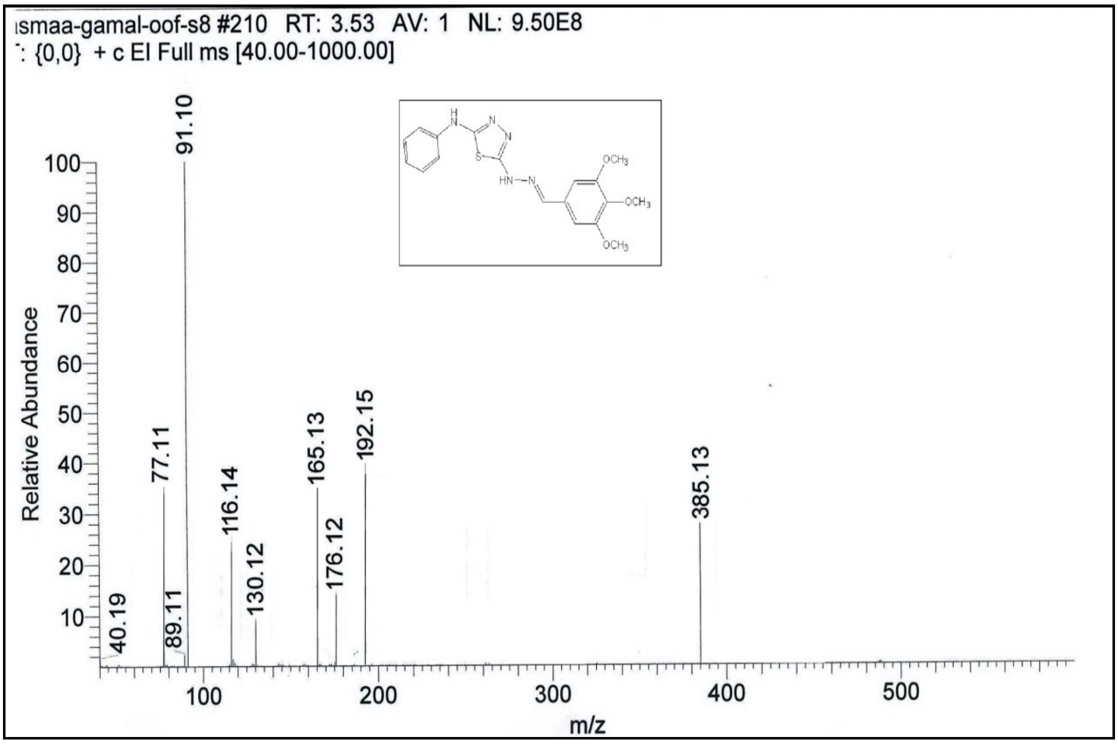

SI Figure S15: Mass spectroscopy of **2d**

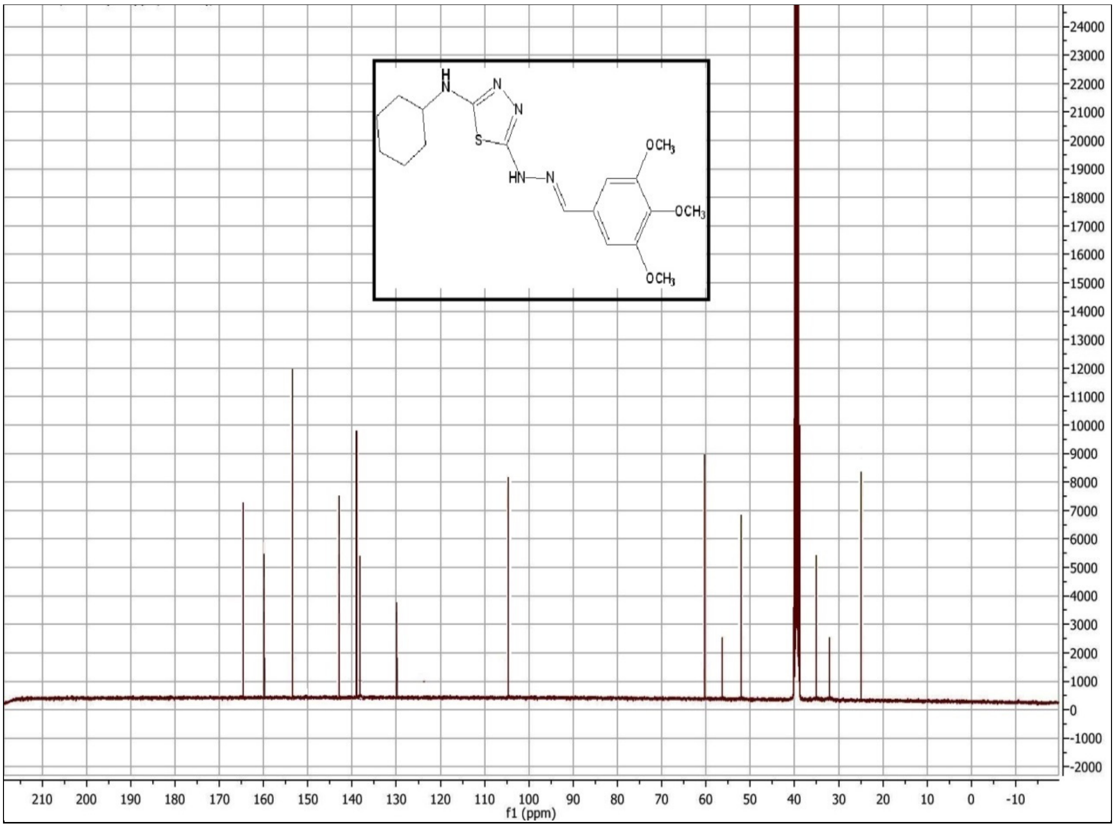

SI Figure S16: ^{13}C-NMR spectrum of **2e**

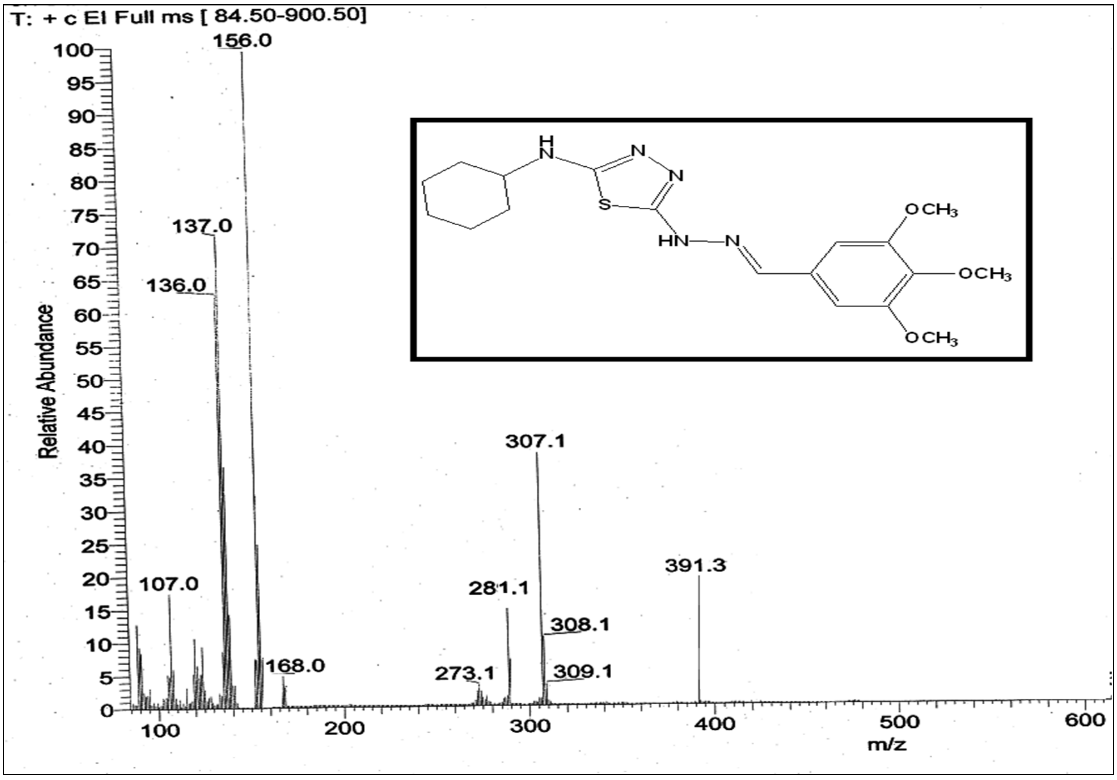

SI Figure S17: Mass spectroscopy of **2e**

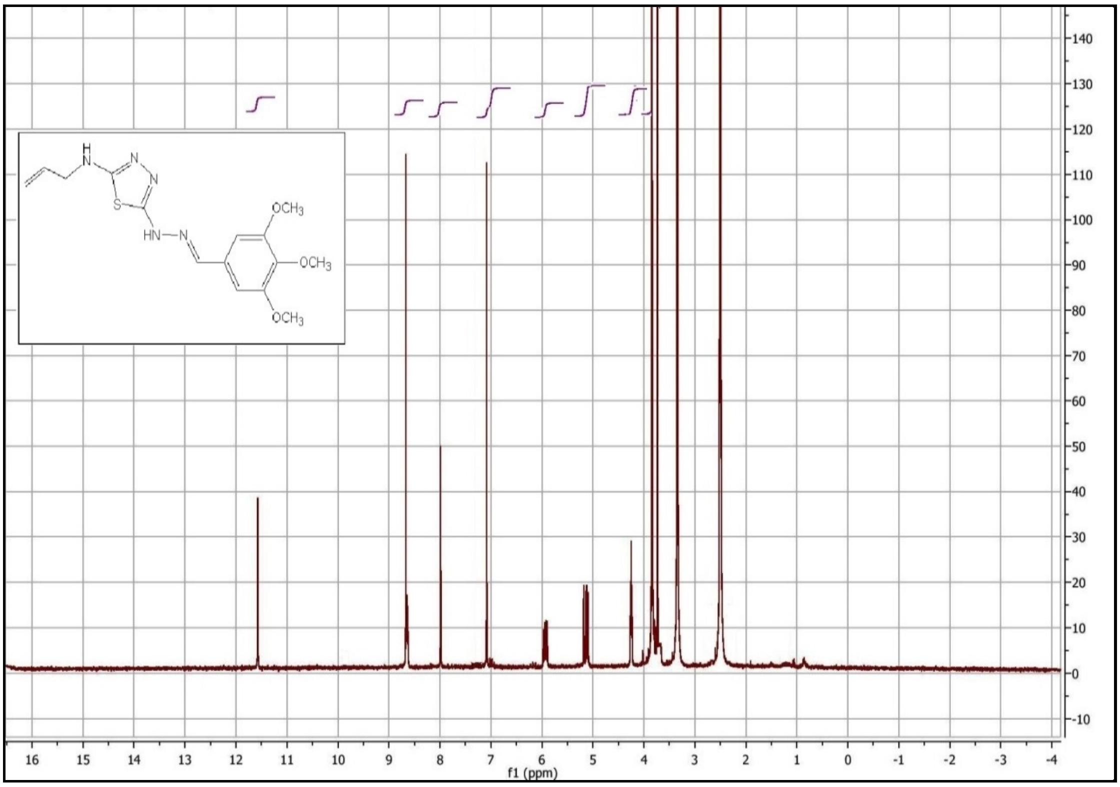

SI Figure S18: ^{1}H-NMR spectrum of **2f**

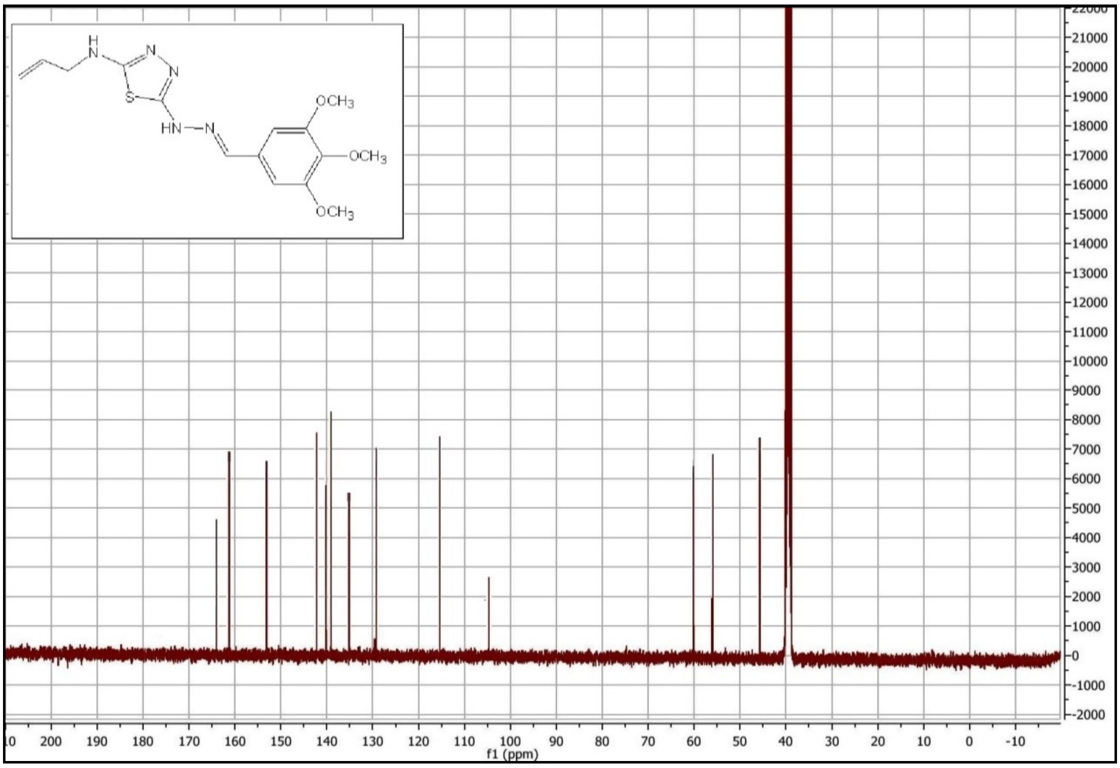

SI Figure S19: ^{13}C-NMR spectrum of **2f**

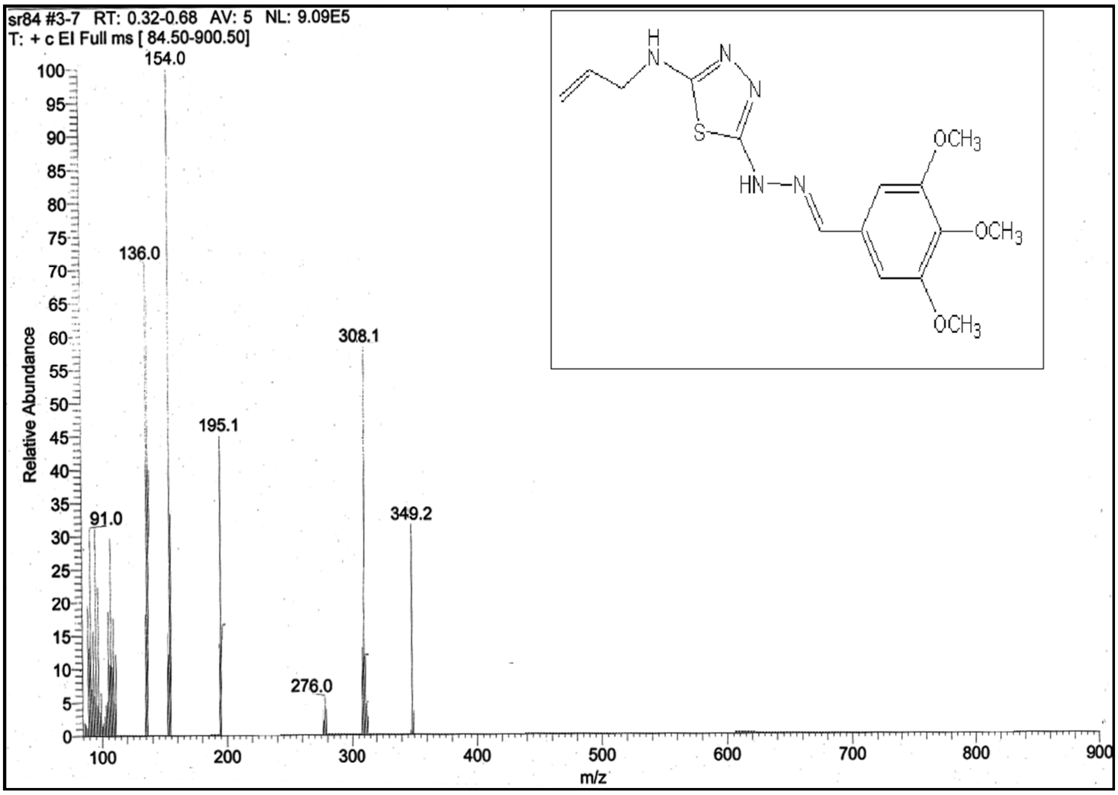

SI Figure S20: Mass spectroscopy of **2f**

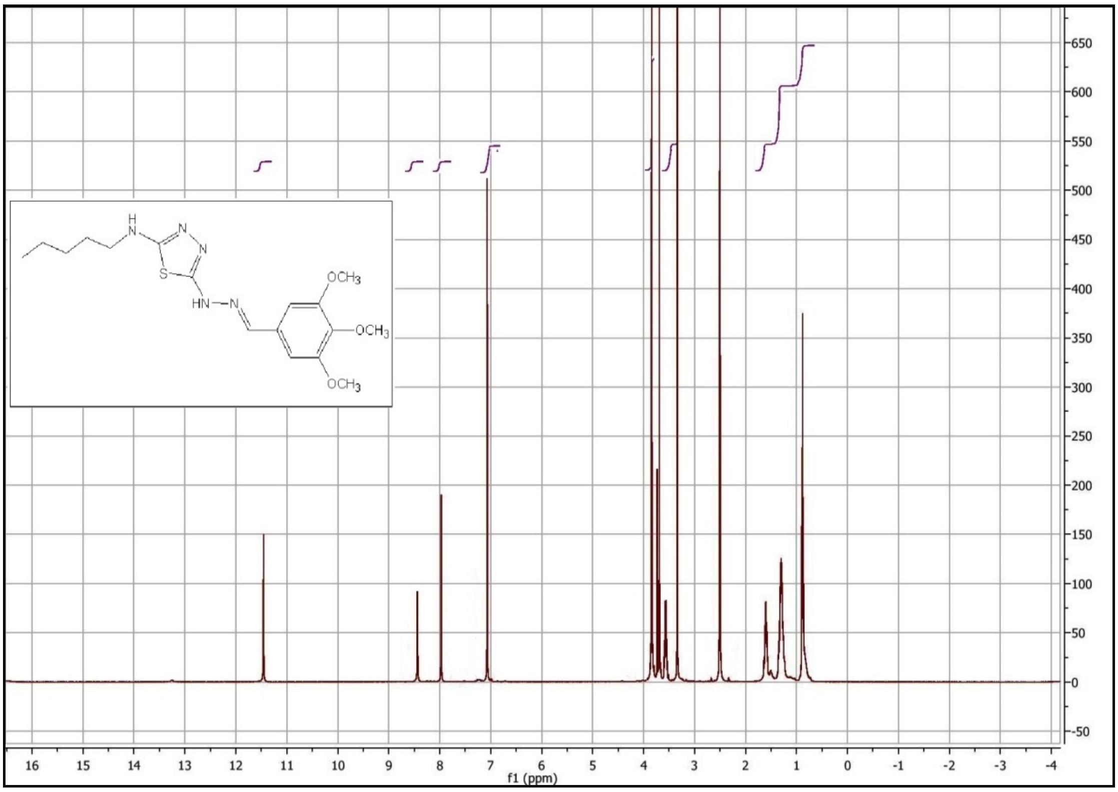

SI Figure S21: ^{1}H-NMR spectrum of **2g**

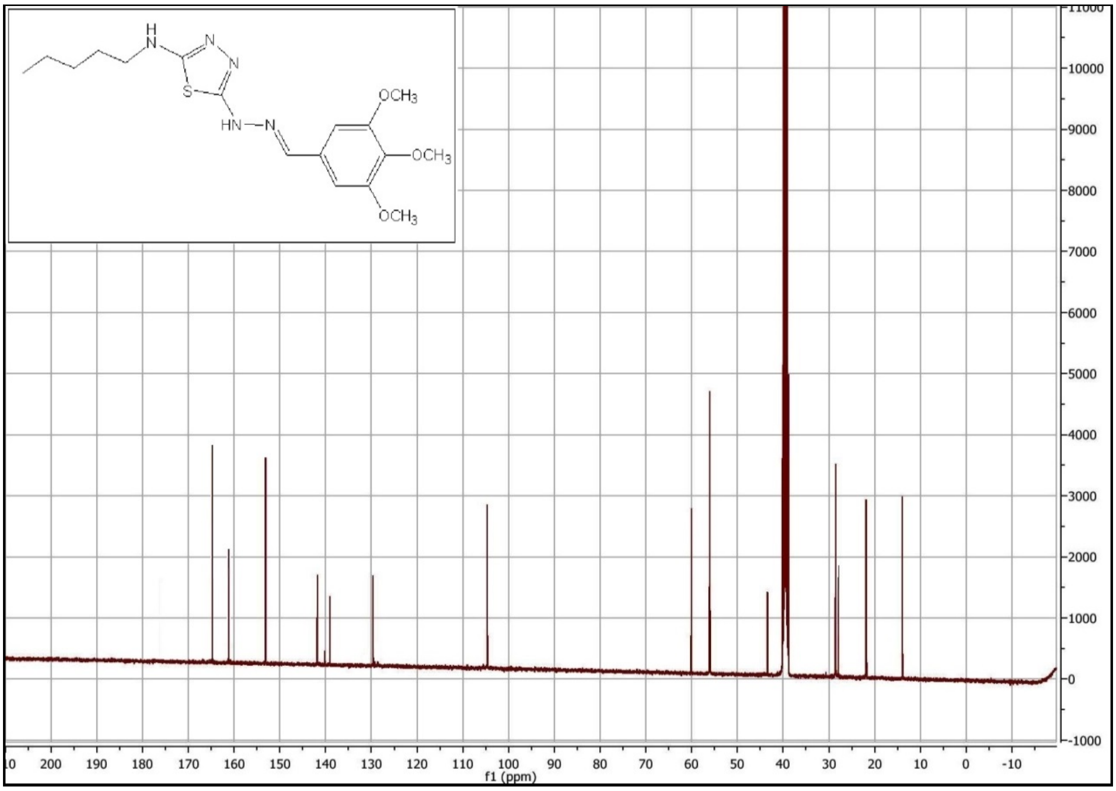

SI Figure S22: ^{13}C-NMR spectrum of **2g**

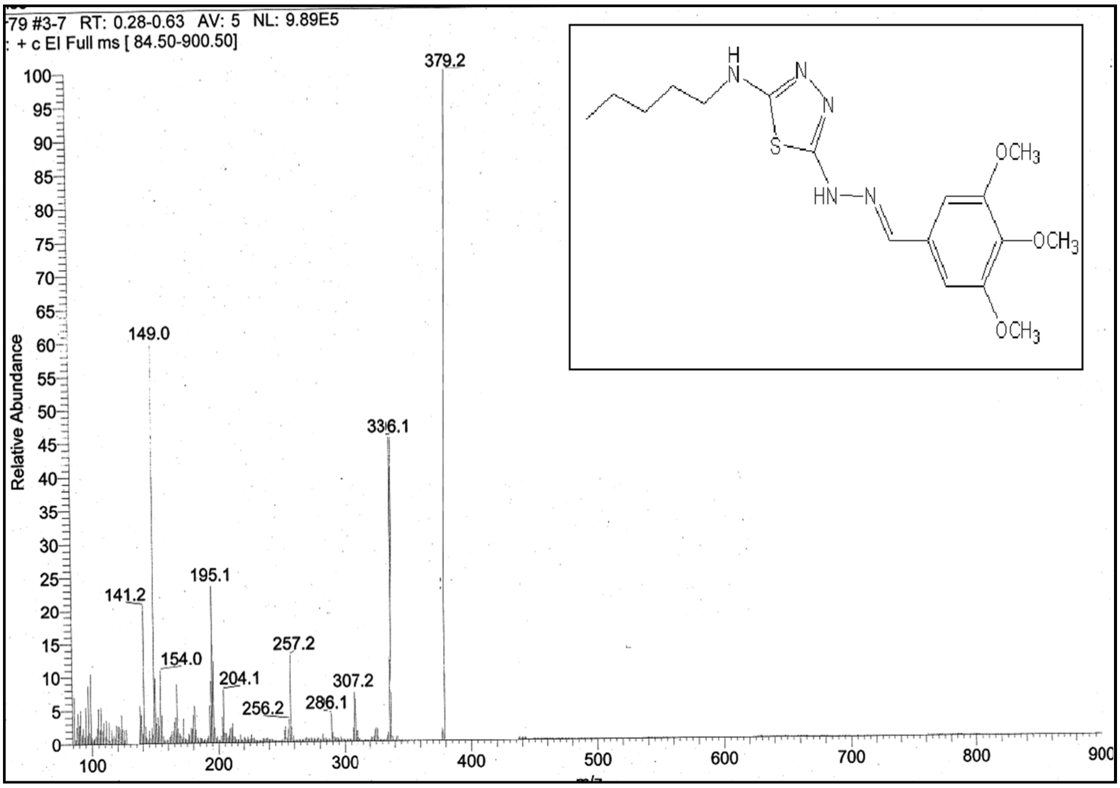

SI Figure S23: Mass spectroscopy of **2g**

Biological Evaluation

2. Results and discussion

Table S8: creatinine, urea and EP concentrations in testis of I/R rats treated with compounds **1a-g** and NAC

Groups	Creatinine (mg/dl)	Urea (mg/dl)	EP (ng/mg protein)
**Control**	0.89± 0.11	39.88±3.13	41.97± 3.92
**NAC**	0.97± 0.11	39.41±3.26	45.90± 3.48
**I/R**	3.18 ± 0.39[ab]	92.17±4.79[ab]	15.18± 1.21[ab]
**1a**	0.98± 0.09[c]	41.99±3.86[c]	42.58± 4.17[c]
**1b**	2.59± 0.19[ab]	93.59±3.57[ab]	13.13± 1.06[ab]
**1c**	1.8± 0.17[c]	58.87±4.12[c]	26.58± 2.567[abc]
**1d**	1.04± 0.10[c]	40.74±4.04[c]	47.41± 2.99[c]
**1e**	1.37± 0.17[c]	43.86±2.89[c]	46.77± 2.08[c]
**1f**	1.89± 0.23[ac]	58.94±4.65[c]	25.06± 2.19[abc]
**1g**	3.41± 0.28[ab]	95.69±4.91[ab]	14.05± 1.64[ab]

Results represent the mean ± S.E. (n=6).
(EP=epinephrine, Nac=N-acetylcystaiene, IR=ischemia reperfusion)
[a] Significant ($P < 0.05$) difference from control group.
[b] Significant ($P < 0.05$) difference from Nac group.
[c] Significant ($P < 0.05$) difference from IR group.

Table S9: Renal MDA, renal NOx and serum TAC concentrations in testis of I/R rats treated with compounds **1a-g** and NAC

Groups	Renal MDA (nmol/gm tissue)	Renal NOx (nmol/gm tissue)	Serum TAC (mmol/l)
**control**	53.00± 3.03	130.40± 4.77	3.20±0.38
**NAC**	54.79± 4.51	129.50± 3.84	3.17±0.30
**I/R**	153.1± 4.76[ab]	56.93± 4.62[ab]	0.89±0.07[ab]
**1a**	54.70± 4.61[c]	130.70± 3.29[c]	3.38±0.37[c]
**1b**	154.6±5.72[ab]	57.42± 3.79[ab]	1.02±0.09[ab]
**1c**	85.48±3.79[abc]	81.78± 3.61[abc]	1.92±0.16[ab]
**1d**	53.34±3.44[c]	126.70± 3.35[c]	2.75±0.23[c]
**1e**	54.96±4.96[c]	128.70± 3.08[c]	3.32±0.24[c]
**1f**	85.34±3.06[abc]	82.68± 4.16[abc]	1.91±0.18[ab]
**1g**	154.0±6.28[ab]	56.37± 4.44[ab]	1.46±0.13[ab]

Results represent the mean ± S.E. (n=6).
(MDA=manoldialdahyde, NOx=total nitrite/nitrate, TAC=total antioxidant capacity, , Nac=N-acetylcystaiene, IR=ischemia reperfusion)
[a] Significant ($P < 0.05$) difference from control group.
[b] Significant ($P < 0.05$) difference from Nac group.
[c] Significant ($P < 0.05$) difference from IR group.

Table S10: creatinine, urea and EP concentrations in testis of I/R rats treated with compounds **2a-g** and NAC

Groups	Creatinine (mg/dl)	Urea (mg/dl)	EP (ng/mg protein)
control	0.88± 0.17	40.22±3.92	40.97± 2.99
NAC	1.00± 0.15	39.41±3.72	44.23± 4.20
IR	3.34 ± 0.38[ab]	96.33±3.55[ab]	13.68± 1.51[ab]
2a	1.15± 0.14[c]	37.66±2.09[c]	40.41± 3.41[c]
2b	3.00± 0.24[ab]	93.95±3.58[ab]	13.463± 1.37[ab]
2c	1.05± 0.14[c]	37.41±2.91[c]	44.51± 3.44[c]
2d	1.85± 0.21[c]	57.04±4.78[c]	26.92± 3.16[abc]
2e	1.19± 0.15[c]	40.74±3.04[c]	43.91± 2.82[c]
2f	2.86± 0.33[ab]	75.53±6.21[ab]	15.10± 1.39[ab]
2g	2.10± 0.22[abc]	57.28±4.83[c]	27.39± 1.83[abc]

Results represent the mean ± S.E. (n=6).
(EP=epinephrine, Nac=N-acetylcystaiene, IR=ischemia reperfusion)
[a] Significant ($P < 0.05$) difference from control group.
[b] Significant ($P < 0.05$) difference from Nac group.
[c] Significant ($P < 0.05$) difference from IR group.

Table S11: Renal MDA, renal NOx and serum TAC concentrations in testis of I/R rats treated with compounds **2a-g** and NAC.

Groups	Renal MDA (nmol/gm tissue)	Renal NOx (nmol/gm tissue)	Serum TAC (mmol/l)
control	52.33± 4.98	130.40± 4.77	3.20±0.38
NAC	51.63± 3.69	129.50± 3.84	3.17±0.30
I/R	161.40± 6.44[ab]	56.93± 4.62[ab]	0.89±0.07[ab]
2a	55.86± 3.19[c]	125.70± 6.02[c]	3.46±2.51[c]
2b	162.30±7.04[ab]	54.75± 5.18[ab]	0.99±0.08[ab]
2c	54.55± 2.80[c]	133.20± 3.51[c]	3.14±0.27[c]
2d	86.31±3.64[abc]	86.78± 5.16[abc]	1.58±0.12[ab]
2e	57.01±2.37[c]	125.10± 2.28[c]	2.91±0.21[c]
2f	155.00±4.95[ab]	53.70± 3.96[ab]	0.98±0.01[ab]
2g	84.01±3.36[abc]	85.57± 4.45[abc]	1.56±0.26[ab]

Results represent the mean ± S.E. (n=6).
(MDA=manoldialdahyde, NOx=total nitrite/nitrate, TAC=total antioxidant capacity, , Nac=N-acetylcystaiene, IR=ischemia reperfusion)
[a] Significant ($P < 0.05$) difference from control group.
[b] Significant ($P < 0.05$) difference from Nac group.
[c] Significant ($P < 0.05$) difference from IR group.

Table S12. Effect of thiadiazole derivative **2c** and its corresponding hydrazinecarbothioamide **1c** relative to NAC on the active Cell-based caspases-3, 8 and 9 in UO-31 cell line.

Caspase type	Control	NAC	1c		2c	
			Conc (ng/mL)	Fold	Conc (ng/mL)	Fold
Casp-3	217±6.1	135.2±3.8	163.1±0.6	1.33	69.77±4.93	3.11
Casp-8	54.38±1.5	11.96±0.8	20.56±2.1	2.64	35.57±1.3	1.53
Casp-9	60.08±2.8	46.83±1.3	50.6±3.4	1.19	17.58±0.65	3.41

Table S13: Molecular docking data for compounds **SR30-37** and NAC in caspase-3 active site (PDB ID 1RHJ).

Code	Docking score/Ki	Interaction with amino acid residue	Distance (°A)	Binding Energy (Kcal/mol)
**NAC**	-3.68	hydrogen bonding (H-donor) with ASP791<br>hydrogen bonding (H-donor) with GLU789<br>hydrogen bonding (H-acceptor) with LYS259	3.65<br>2.80<br>3.08	-1.1<br>-5.0<br>-0.5
**2a**	-36.84	hydrogen bonding (H-donor) with ASP791<br>Hydrophobic bonding (pi-H) with ARG786<br>Hydrophobic bonding (pi-H) with ARG786	3.16<br>3.81<br>4.41	-1.9<br>-1.8<br>-0.6
**2b**	-39.77	hydrogen bonding (H-acceptor) with LYS259<br>Hydrophobic bonding (pi-H) with ASP791	3.18<br>4.32	-3.4<br>-0.6
**2c**	-35.42	hydrogen bonding (H-donor) with CYS792<br>Hydrophobic bonding (pi-H) with ASP791	4.12<br>3.94	-2.1<br>-1.3
**2d**	-35.28	Hydrophobic bonding (pi-H) with ARG266	4.31	-0.7
**2e**	-32.13	Hydrophobic bonding (pi-H) with ARG266	4.03	-0.8
**2f**	-24.58	hydrogen bonding (H-acceptor) with LYS259<br>Hydrophobic bonding (pi-H) with ARG266	2.95<br>4.04	-4.0<br>-0.8
**2g**	-32.39	Hydrophobic bonding (pi-H) with ARG266	4.50	-0.6

3. Experimental

3.2. Biology

5.1. **Materials and methods of biomarkers (creatinine, urea, Ep, MDA, TAC, NOx)**

- Urea and creatinine kits (Diamond Diagnostics, Egypt).

-TAC kit (Biodiagnostics, Egypt).

- **Determination of serum creatinine**

Serum creatinine was measured using an enzymatic colorimetric creatinine kit (Schirmeister, 1964).

Principle

The assay was based on the reaction of creatinine with sodium picrate. Creatinine reacts with alkaline picrate forming a red complex. The time interval chosen for measurements avoided interferences from other serum constituents. The intensity of the color formed was proportional to the creatinine concentration in the sample.

Reagents

- **R1:** Creatinine standard (2 mg/dl).
- **R2:** Picric acid 17.5 mmol.
- **R3:** Sodium hydroxide 0.29 mol/l.

Procedure

- An aliquot of 0.50 ml of the working reagent was added to each of the reagent blank, sample and standard test tubes.
- Add 0.25 ml distilled water and 0.25 ml of trichloroacetic acid to blank tube only.
- Then, 0.50 ml from the sample as well as 0.25 ml of standard was added to their corresponding tubes and 0.25 ml of trichloroacetic acid was added only to the standard tube. They were mixed and incubated for 20 min at room temperature.
- The absorbance of the unknown and the standard against reagent blank was measured at 546 nm, within 20 min using Bechman DU-64 spectrophotometer.
- Values are derived by means of the following calculations:
  - Serum Creatinine (mg/dl) = $\frac{A_u}{A_s} * Cs$

Where Au and As are the absorbance values of the sample and standard, respectively, and Cs is the concentration of the standard (mg/dl) which equal to 2. The steps were the same for determination of the creatinine in urine except that the dilution of the sample by 1/50 with distilled water then multiply results by 50 (dilution factor).

-Determination of serum urea

Reagents

- **R1:** Urea aqueous primary standard 50 mg/dL.
- **R2:** Urease.
- **R3:** When reconstituted the reagent contains the following: phosphate buffer, sodium salicylate, sodium nitro-prusside, and EDTA.
- **R4:** Sodium hydroxide and sodium hypochloride.

Procedure

- An aliquot of 1 ml of the R3 was added to each of the reagent blank, sample and standard test tubes followed by one drop of R2. Then, 10 µl from the sample as well as the standard were added to their corresponding tubes.
- They were mixed well and incubated for 3 min at room temperature.
- Then, 200 µl of R4 was added to each of the reagent blank, sample and standard, mixed well and incubated 10 min at room temperature.
- The absorbance (A) of the unknown and the standard against reagent blank was measured at 578 nm, within 60 min, using Bechman DU-64 spectrophotometer, USA.
- Values are derived by means of the following calculations:

$$\text{Urea (mg/dl)} = \frac{A_u}{A_s} * 50$$

where Au and As are the absorbance values of the samples and standard, respectively, and 50 is the concentration of the standard (mg/dl).

Assessment of total nitrite in kidney

Procedure

- Add an aliquot of 250 µl of sample to a test tube.
- 1000 µl of glycine buffer was added to each tube.
- Add copperized cadmium granules (0.75 g of Cd granules to each tube).
- The tubes were then incubated at room temperature for 1 h with thorough shaking.
- The reaction was stopped by the addition of 250 µl of 0.35 M sodium hydroxide.
- An aliquot of 1000 µl of 120 mM zinc sulfate solution was added under vortex and the solution was allowed to stand for 10 min.
- The tubes were centrifuged at 4000*g* for 10 min.
- Transfer an aliquot of 1000 µl of the clear supernatant to another test tube and 500 µl of 1.0% sulfanilamide (prepared in 3 N HCl) and 500 µl 0.1% *N*-naphthylethylenediamine (prepared in water) were added with shaking.
- The absorbance was measured at 545 *n*m against a blank containing the same concentration of ingredients after 10 min, but no biological sample using Bechman DU-64 spectrophotometer.
- A standard curve was constructed with a set of serial dilutions (from 10 *n*mol/L to 1 mmol/L) of sodium nitrite. From this curve, the total nitrite content in the unknown sample was extrapolated from the corresponding absorbance using the regression line from the standard curve and expressed as *n*mol/g tissue.

Activation of cadmium

- Cadmium reagent was prepared by washing with distilled water, discarding the supernatant.
- Wash twice with 0.5 N HCl. At this point the cadmium texture resembled stones resting on the bottom of the flask.
- Then, cadmium was washed twice with distilled water and then washed with 5% copper sulphate to coat the cadmium with copper. The wash with copper sulphate was repeated two to three times until the solution above the cadmium remained blue for 60 seconds to ensure that the cadmium was saturated with copper. Change of the color of cadmium to red or black means that it became unusable and discarded.
  - Once the cadmium was saturated with copper, the excess copper was removed quickly by rinsing with four washes of distilled water.
  - The copper-coated cadmium was then washed twice with 30 ml of 0.1 N HCl and stored in this HCl solution ready for use. The copper-coated cadmium appeared gray [34]

Histopathological Investigation

- Photography

An Olympus light microscopy and digital camera (Olympus, Markham, ON, Canada), were used in this study. Images were executed using Adobe Photoshop.

- Morphometric Study

Semi-quantitative data were measured and counted per section in ten randomly selected non-overlapping fields using power × 400 magnifications of the sections from each rat [36]. The results were carried out using the Image J program analysis software (Image J 1.48V, Maryland, MD, *USA;* Wayne Rasb and National Institutes of Health).

- Statistical Analysis

Data were evaluated as means (standard error of the mean). One-way analysis of variance (ANOV) with the use of Turkey's post-test was carried out for the analysis of the results to detect the significant difference statistically. *P* values < 0.05 were considered significant.

Molecular Docking Study

The target compounds were constructed into a 3D model using the builder interface of the MOE program and docked into the active site of caspase-3 (PDB: 3GJQ). Checking their structures and the formal charges on atoms by 2D depiction was carried out and the energy, was minimized until an RMSD (root mean square deviations) gradient of 0.01 Kcal/mol and RMS (Root Mean Square) distance of 0.1 A with MMFF94X (Merck molecular force field 94x) force-field and the partial charges were automatically calculated
